# AcrB Trimer Stability and Efflux Activity, Insight from Mutagenesis Studies

**DOI:** 10.1371/journal.pone.0028390

**Published:** 2011-12-05

**Authors:** Linliang Yu, Wei Lu, Yinan Wei

**Affiliations:** Department of Chemistry, University of Kentucky, Lexington, Kentucky, United States of America; University of Massachusetts, United States of America

## Abstract

The multidrug transporter AcrB in *Escherichia coli* exists and functions as a homo-trimer. The assembly process of obligate membrane protein oligomers, including AcrB, remains poorly understood. In a previous study, we have shown that individual AcrB subunit is capable of folding independently, suggesting that trimerization of AcrB follows a three-stage pathway in which monomers first fold, and then assemble. Here we destabilized the AcrB trimer through mutating a single Pro (P223) in the protruding loop of AcrB, which drastically reduced the protein activity. We replaced P223 separately with five residues, including Ala, Val, Tyr, Asn, and Gly, and found that AcrB_P223G_ was the least active. Detailed characterization of AcrB_P223G_ revealed that the protein existed as a well-folded monomer after purification, but formed a trimer *in vivo*. The function of the mutant could be partly restored through strengthening the stability of the trimer using an inter-subunit disulfide bond. Our results also suggested that the protruding loop is well structured during AcrB assembly with P223 served as a “wedge” close to the tip to stabilize the AcrB trimer structure. When this wedge is disrupted, the stability of the trimer is reduced, accompanied by a decrease of drug efflux activity.

## Introduction

The inherit difficulty of expression and purification of membrane proteins has drastically hindered studies of these important players of cellular functions. In the past decade, there has been a leap in the effort of solving crystal structures of membrane proteins. As of Jun. 2011, there are almost 300 unique structures of membrane proteins in the protein data bank. The availability of an increasing number of protein structures has set the stage for studies of the dynamic life cycles of membrane proteins, starting from the folding and assembly of nascent polypeptide chains in the membrane that leads to functional proteins. Specifically, the assembly process of obligate homo-oligomeric membrane proteins remains elusive [1]–[3]. Obligate oligomers exist and function exclusively in their oligomeric form. However, it was not clear how multiple subunits, after their co-translational membrane insertion, assemble into the final functional state. Toward answering these questions, we chose an *Escherichia coli* inner membrane protein AcrB as a model system to study its oligomerization.

**Figure 1:**
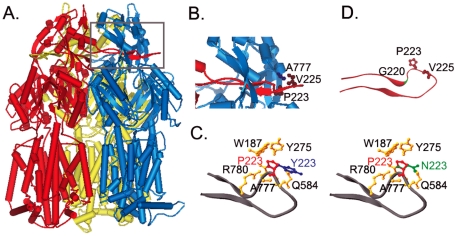
Crystal structure of AcrB. **A.** AcrB trimer with each subunit color coded (created from 2HRT.pdb). **B.** Zoom in view of the loop region (grey box in A). Residues P223 and V225 from the red subunit, and A777 from the blue subunit are highlighted using ball-and-stick models. **C.** Binding pocket of P223 (red). Residues that form the binding pocket of P223 were shown (orange). The conformations of Y223 (blue) and N223 (green) were also shown superimposed on top of P223. **D.** Ribbon diagram of the protruding loop at a different angle. Residues P223 and V225 are highlighted using ball-and-stick models. Position of G220 is highlighted in green.

AcrB is an obligate homo-trimer. It associates with the peripheral protein AcrA and outer membrane protein TolC to form a complex that spans from the cytoplasm all the way to the exterior of the cell [4]–[7]. AcrAB-TolC and its homologues, members of the resistance-nodulation-cell division (RND) transporter family, are major efflux systems that make Gram-negative bacteria resistant against a wide range of cytotoxic compounds [8], [9]. The structure of AcrB has been solved by x-ray crystallography in both the apo and substrate-bound conformations [10]–[15]. Based on the crystal structure of AcrB, a conformational cycling model for drug transport has been proposed [16]–[19]. However, crystal structures can not provide insight into the biogenesis process of an AcrB trimer. Recently, we have created a monomeric AcrB mutant, AcrB_Δloop_, in which we deleted 17 residues from a protruding loop [20] (Figure 1). The loop is obviously important for inter-subunit interactions, as it penetrates deep into a tunnel in the neighboring subunit. While at the same time, it stretches away from the rest of the polypeptide chain, not making tertiary contact with any residues from the same subunit. We found that AcrB_Δloop_ completely lost its transport activity and failed to assembly into a trimer, while had a similar tertiary structure as subunits in the AcrB trimer. These results indicated that monomeric AcrB was capable of folding independently, suggesting that oligomerization of AcrB occurred through a three-stage pathway, in which nascent polypeptide chains first folded independently into monomers, which then assembled into functional trimers.

To further probe the role and structural flexibility of the protruding loop during AcrB trimerization, we mutated a conserved Pro (P223) and characterized the structure and function of the resultant mutants. We found that replacing P223 with other residues drastically decreased the stability of the AcrB trimer and caused a loss of function, which could be regained partially through connecting subunits in a trimer covalently using a disulfide bond.

## Results

### Effect of P223 mutation on AcrB drug efflux activity

**Figure 2:**
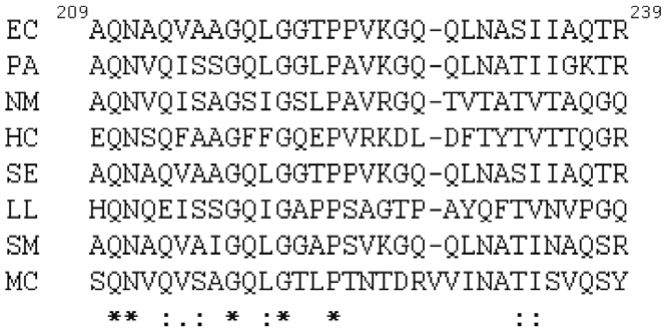
Sequence alignment of the loop. The numbers indicate positions of the starting and ending residues in the sequence of *E. coli* AcrB. Asterisks, colons and periods indicate identical, conserved and semi-conserved residues, respectively. The sequences are: EC, AcrB from *E. coli*; PA, MexB from *Pseudomonas aeruginosa PAO1*; NM, MtrD from *Neisseria meningitidis 8013*; HC, hypothetical protein HcanM9_00968 from *Helicobacter canadensis MIT 98-5491*; SE, acridine efflux pump from *Salmonella enterica subsp. enterica serovar Typhimurium str. LT2*; LL, acriflavine resistance protein B from *Legionella longbeachae D-4968*; SM, hydrophobe/amphiphile efflux-1 (HAE1) from *Tenotrophomonas maltophilia R551-3*; MC, RND system efflux pump AcrB from *Moraxella catarrhalis RH4*.

The protruding loop of AcrB is composed of 30 residues, which form two short anti-parallel β-strands in the middle (Figure 1). There are two consecutive Pro in the loop, P223 and P224. Sequence alignment with other AcrB homologues revealed that while P223 was conserved in all sequences, P224 was not (Figure 2). Based on the crystal structure, P223 locates close to the tip of the loop, where it induces the loop to form a kink (Figure 1C and 1D). Pro is unique among twenty common amino acids in that its backbone conformation is much more rigid and can only assume a very limited set of ϕ and ψ angles. The rigidity of Pro has been exploited by many proteins to serve specific structural and functional roles [21]–[24]. P223 seemed to be playing an important role in maintaining the specific structure of the loop and making inter-subunit interaction in AcrB. To examine if this is the case, we created several single mutations, in which P223 was replaced with residues of various size, shape, and polarity, including Gly (AcrB_P223G_), Ala (AcrB_P223A_), Val (AcrB_P223V_), Tyr (AcrB_P223Y_), and Asn (AcrB_P223N_).

To examine the effect of these mutations on the function of AcrB, we tested the minimum inhibitory concentration (MIC) of the mutants to five established AcrB substrates [25] (Table 1). As described in the materials and methods session, plasmids encoding different AcrB mutants were transformed into an *acrB* gene knockout *E. coli* strain, BW25113*ΔacrB*, for activity assay. The same strain transformed with a plasmid encoding wild type (WT) AcrB or an empty vector without the *acrB* gene (pQE70) were used as positive and negative controls, respectively. As shown in Table 1, when replaced with different residues, all P223 mutations drastically decreased the activity of the efflux pump. Among these mutations, P223G had the most drastic detrimental effect on protein activity. The effects of P223V and P223Y were comparable, which were greater than the effects of P223A and P223N. A comparison of the effects of different mutations revealed that the extent of disruption of AcrB function did not correlate with the size of the side chains. P223 locates close to the tip of the long loop (Figure 1B). The binding pocket of P223 is semi-open, formed by residues W187, Y275, Q584, A777, and R780 from the neighboring subunit. We created structure models of AcrB_P223Y_ and AcrB_P223N_ using the online server of SWISS-MODEL (Figure 1C) [26]. As shown in Figure 1C, the bulky side chain of Tyr and Asn rotated out of the binding pocket and was partially exposed. Therefore, little structural rearrangement would be necessary to accommodate mutations of P223. The energetic penalty associated with exposing a hydrophobic side chain could be the reason why P223Y and P223V were the least active mutants among all five. Backbone flexibility seemed to play a very important role, since P223G, which introduced the largest increment of backbone flexibility, was the least active. In terms of the neighboring and non-conserved residue P224, the replacement with Gly had no observable effect on protein function (Table 1).

**Table 1:** MIC (µg/ml) of BW25113*ΔacrB* containing plasmid encoded AcrB.

Plasmids	Ery*	Nov	R6G	TPP	Tet
pQE70-AcrB	80	160–320	320–640	640	1.28
pQE70	2.5	5	5	5	0.32
pQE70-AcrB_P224G_	80	160	320	320	1.28
pQE70-AcrB_P223G_	5	10	20	20	0.32
pQE70-AcrB_P223A_	10	40	80	40	0.64
pQE70-AcrB_P223V_	5	20	40–80	40	0.64
pQE70-AcrB_P223Y_	5	20	40–80	20	0.64
pQE70-AcrB_P223N_	5	40	80	40	0.64
pQE70-AcrB_V225C_	80	160	320	640	1.28
pQE70-_CL_AcrB_A216C/I234C_	80	160	640	320	1.28
pQE70-_CL_AcrB_P223G/A216C/I234C_	5	10	20	10	0.32
pQE70-_CL_AcrB_P223G/V225C_	2.5	5	5	5	0.32
pQE70-_CL_AcrB_P223G/V225C/A777C_	40	40	160	80	0.64
pQE70-_CL_AcrB_V225C/A777C_	80	160	320	640	1.28

*Drugs tested were Erythromycin (Ery), Novobiocin (Nov), Rhodamine 6G (R6G), Tetraphenylphosphonium (TPP), and Tetracycline (Tet).

### Structural Characterization of AcrB_P223G_

**Figure 3:**
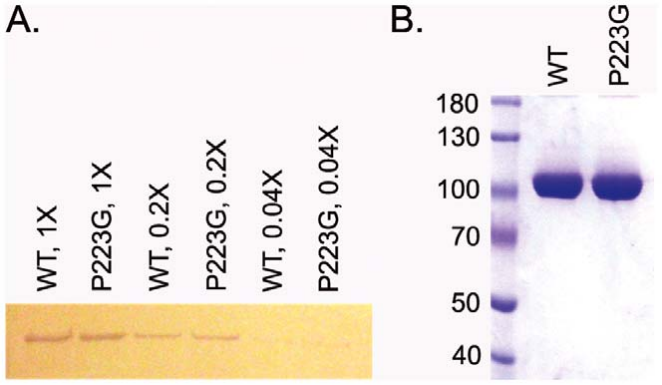
Comparison of the expression levels of WT AcrB and AcrB_P223G_. **A.** Western blot analysis of membrane vesicles extracted from BW25113Δ*acrB* expressing WT AcrB (WT) or AcrB_P223G_ (P223G). Each sample was diluted 1, 5, and 25 folds. **B.** SDS-PAGE analysis of purified WT AcrB and AcrB_P223G_. The expression levels of the WT and AcrB_P223G_ are similar.

We chose AcrB_P223G_ for further characterization, since it had the most dramatic effect on the function of AcrB. To determine the effect of P223 mutation on the protein expression level, we extracted membrane vesicles from BW25113Δ*acrB* expressing WT AcrB or AcrB_P223G_ and conducted quantitative Western blot analysis using an anti-AcrB antibody (Figure 3A). There was no significant difference between the expression levels of WT AcrB and AcrB_P223G_, indicating that the increase of drug susceptibility was not due to a decrease of protein expression level.

**Figure 4:**
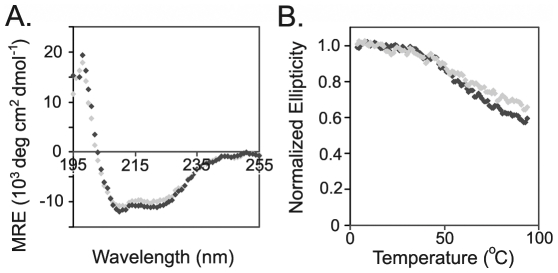
CD spectra of purified WT AcrB and AcrB_P223G_. **A.** Wavelength scans at the far UV region of WT AcrB (black) and AcrB_P223G_ (grey) superimposed well onto each other, indicating that the two proteins had similar secondary structure contents. **B.** Temperature denaturation curves of WT AcrB (black) and AcrB_P223G_ (grey). The ellipticity values monitored at 222 nm were normalized to the reading at 4°C. The thermal stabilities of the two proteins were similar.

AcrB_P223G_ could be purified with similar yield and purity as WT AcrB, as shown in Figure 3B. We collected far UV circular dichroism (CD) spectra of both proteins to compare their secondary structure compositions (Figure 4A). Both proteins had high α-helical contents, consistent with the crystal structure of AcrB. The two spectra overlapped reasonably well, indicating that the secondary structure of the mutant was similar to that of the WT protein.

Thermal denaturation is a useful experiment in determining the stability of membrane proteins *in vitro* [27], [28]. With the slow increase of temperature, the purified proteins gradually lost their helical secondary structures as monitored via the change of ellipticity at 222 nm (Figure 4B). Approximately 40% of helical content in the protein was lost when the temperature increased from 4 to 98°C for both WT AcrB and AcrB_P223G_, which was consistent with earlier observations [20]. The melting curves of the two proteins were close to each other, with the ellipticity for WT AcrB slightly lower at 98°C.

**Figure 5:**
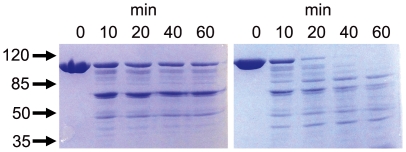
Limited trypsin digestion of purified WT AcrB (left) and AcrB_P223G_ (right). Lanes 2 to 5 were samples taken at 10, 20, 40, and 60 min into the digestion, respectively. Lane 1 was a control sample in which no trypsin was added. WT AcrB was digested slower than AcrB_P223G_.

Limited proteolysis has been used widely to reveal the presence of compact domains, assess the structural flexibility, and examine the topology of proteins [29]–[31]. In this study, we digested WT AcrB and AcrB_P223G_ using trypsin under mild conditions to compare their structural differences. WT AcrB was more resistant to trypsin digestion than AcrB_P223G_ (Figure 5). Under our experimental condition, AcrB_P223G_ was completely digested to small fragments in 40 minutes, while WT AcrB retained a decent amount of full length protein during the same digestion period. As a negative control, we have also digested a fully unfolded AcrB as previously described [20]. Unfolded AcrB was completely digested under the experimental condition within 5 min (data not shown).

The proteolysis results indicated that in AcrB_P223G_, trypsin recognition sites (Arg and Lys) were more exposed compared to the same residues in WT AcrB. However, they were much more protected than digestion sites in fully unfolded AcrB. Two structural changes could have caused this effect. First, P223 to Gly mutation could have changed the tertiary structure of the protein to make it less compactly folded. Under this condition, potential trypsin digestion sites will be less protected than similar sites in a more compactly folded structure. Second, the mutation could have caused AcrB trimer to dissociate, under which condition the inter-subunit interface, which was protected in an AcrB trimer, would be exposed upon trimer dissociation [20].

**Figure 6:**
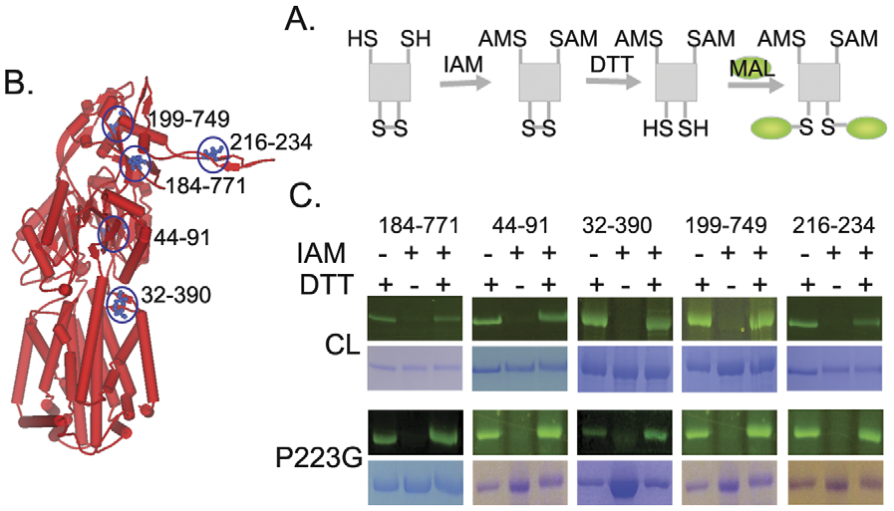
Disulfide trapping analysis of AcrB tertiary structure. **A.** Schematic illustration of the blocking-reducing-labeling procedure. **B.** The locations of reporter Cys pairs in the structure of AcrB were highlighted using black circles and blue ball-and-stick models. Residue numbers of the Cys mutations were marked. **C.** AcrB tertiary structure as revealed by the disulfide trapping method. The extents of disulfide bond formation for each reporter Cys pair were very similar in AcrB_P223G_ as compared to WT AcrB. Therefore, the overall conformation, or tertiary structure, of AcrB_P223G_ was very similar to that of WT AcrB.

To characterize the tertiary structure of AcrB_P223G_, we used a disulfide-trapping method developed earlier in our laboratory [32]. We have previously identified seven pairs of residues in the periplasmic domain of AcrB that are within the disulfide bond distance. Mutations of these residues to Cys and formation of disulfide bonds at these positions have little effect on the function of AcrB. We have used these Cys-pair mutants as reporters to investigate the tertiary structure of AcrB, in which the formation of disulfide bond could be detected through labeling with a fluorescent probe. If the structure of a mutant is similar to that of WT AcrB, we expect to see a similar fluorescent labeling profile for the Cys pair reporters in both proteins. Figure 6A showed the concept of detecting disulfide bond using fluorescent labeling. Briefly, protein was purified in the presence of iodoacetamide (IAM), which blocked free thiols but did not affect disulfide bond. After purification, protein samples were reduced using dithiothreitol (DTT), and then labeled using a thiol-specific fluorescent probe, fluorescein-5-maleimide (Flu-MAL). Under this experimental condition, only proteins that contain disulfide bond in their native structure would be labeled and fluoresce (third lane in each gel picture in Figure 6C). In each experiment, we have also included internal positive and negative controls (first and second lanes in each gel picture in Figure 6C, respectively). In the positive control, proteins were purified in the absence of IAM and then reduced with DTT before labeling. Therefore, all proteins that contained Cys, either in the free thiol or disulfide bond form, would be labeled. In the negative control, IAM was present during protein purification, but the purified samples were not reduced before labeling. Therefore, no protein should be labeled.

We used 4 Cys-pair reporters established previously to evaluate the tertiary structure of AcrB_P223G_ (Figure 6B). In addition, we have created a new reporter Cys pair in this study, A216C–I234C. This pair exists in the loop. We used it to detect potential conformational changes in the loop. We have confirmed that disulfide bond formed between C216 and C234 when they were introduced into a Cys-less AcrB (_CL_AcrB) construct, in which the two intrinsic Cys in the sequence of AcrB were replaced with Ala. In addition, the mutations and formation of disulfide bond had no effect on the drug efflux activity of AcrB (Table 1). Two pictures of SDS-PAGE gels were shown for each protein, one visualized under fluorescence light without staining and the other stained with coomassie blue dye. Coomassie blue stain revealed the loading amount in each lane, while the fluorescence image reflected the formation of disulfide bond. As discussed above, the first two lanes were positive and negative controls, respectively. The third lane revealed the presence of disulfide bond. For each reporter Cys pair tested in the presence of P223G mutation, the fluorescence in the third lane was comparable to that of the WT AcrB, indicating that the disulfide formed similarly as in the WT protein. Furthermore, the new reporter Cys pair, 216–234, showed that the structure of the loop did not change significantly compared to that of the WT protein. These results suggested that the P223G mutation did not change the tertiary structure of the protein.

### AcrB_P223G_ Exists as Monomer *in vitro* and Forms Trimer *in vivo*

**Figure 7:**
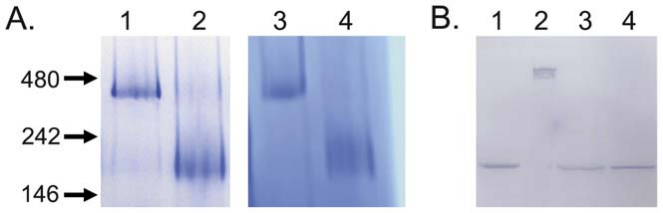
Quaternary structure analyses. **A.** BN-PAGE analyses of freshly purified WT AcrB (lane 1), AcrB_P223G_ (lane 2), _CL_AcrB_P223G/A225C/V777C_ in the absence of DTT (lane 3), and _CL_AcrB_P223G/A225C/V777C_ in the presence of 4 mM DTT (lane 4). Positions of molecular markers were marked on the left of the gel (in kD). A 4–20% gradient gel was used in this experiment. **B.** Western blot analysis of membrane vesicles extracted from BW25113*ΔacrB* expressing WT AcrB (lanes 1 and 3) or _CL_AcrB_P223G/A225C/V777C_ (lanes 2 and 4) in the absence (lanes 1 and 2) or presence (lanes 3 and 4) of 4 mM DTT.

To evaluate the quaternary structure of AcrB_P223G_, we analyzed purified AcrB_P223G_ using blue native (BN)-PAGE. BN-PAGE is a powerful, convenient and inexpensive technique to determine the oligomeric state of membrane proteins and identify physiological protein-protein interactions [33], [34]. It has been used to confirm that the oligomer state of wild-type AcrB as a trimer [18]. As shown in Figure 7A, WT AcrB migrated as a trimer as expected, while AcrB_P223G_ migrated as a monomer. The BN-PAGE result indicated that purified AcrB_P223G_ existed as monomer, which explained its decreased tolerance to trypsin digestion.

Since AcrB_P223G_ had a low level of residual activity, we speculated at least a small portion of the protein should exist as trimer *in vivo*, since AcrB function exclusively as trimers (Table 1). To trap trimers formed in the membrane, we introduced two Cys mutations in AcrB_P223G_, V225C and A777C (Figure 1B). The intrinsic Cys in AcrB was removed to avoid interference. Seeger et al. have shown that C225 and C777 formed inter-subunit disulfide bond when introduced into the sequence of AcrB, which covalently connected subunits in an AcrB trimer without affecting protein function [35]. We extracted membrane vesicles from BW25113*ΔacrB* expressing _CL_AcrB_P223G/V225C/A777C_ in the presence of IAM and subjected the sample to SDS-PAGE and Western blot analysis with or without the addition of DTT (Figure 7B). In the absence of DTT, a clear band is visible at the high molecular weight range, indicating the protein formed trimers *in vivo*. In the presence of DTT, the high molecular weight species dissociated to form monomers. BW25113*ΔacrB* expressing WT AcrB was used as a control. Only monomer band was visible both in the presence and absence of DTT. Next, we also analyzed purified _CL_AcrB_P223G/V225C/A777C_ using BN-PAGE (Figure 7A). The protein migrated as trimers in the absence of DTT, which could be reduced into monomers in the presence of DTT. These results suggested that the C225–C777 disulfide bond effectively trapped _CL_AcrB_P223G/V225C/A777C_ trimers. Since AcrB trimer makes direct contact with AcrA, we further examined if the absence of AcrA would affect the efficiency of disulfide bond formation between C225–C777. We expressed both _CL_AcrB_V225C/A777C_ and _CL_AcrB_P223G/V225C/A777C_ in *E. coli* strain AG100A, which is deficient in both *acrA* and *acrB* genes. We found that the efficiency of disulfide bond formation in both proteins were not affected, indicating that the presence of AcrA was not necessary to trap the AcrB trimer (data not shown). The capability of the protein to form trimer in the cell membrane further confirmed the structural characterization results discussed above, in which we found that purified AcrB_P223G_ had similar secondary and tertiary structures to WT AcrB.

Since AcrB functions in the trimeric form, we examined if the introduction of V225C and A777C mutation enhanced the function of AcrB_P223G_ (Table 1). We first introduced the V225C mutation into AcrB_P223G_ to create AcrB_P223G/V225C_. V225 is in the loop, close to P223. We found that this mutation had a slightly detrimental effect on the activity of the mutant and further decreased the MIC for some substrates. To further examine the effect of V225C mutation on the activity of AcrB, we introduce the mutation into a wild type AcrB background. We found that the V225C mutation had little effect on AcrB activity (Table 1). We speculate that V225C mutation may have a small destabilizing effect on AcrB trimer. However, the effect alone was too small to cause an observable change of activity. When introduced into a P223G mutant, the combined effect was larger than the impact of P223G alone and thus caused a bigger drop of activity. Next, we introduced A777C mutation. The V225C/A777C double mutation partially restored the function loss caused by P223G mutation and increased the MICs for several substrates to levels close to that of the wild type protein, suggesting that the formation of disulfide bond between C225 and C777 of neighboring subunits might have stabilized the AcrB trimer and largely restored its activity.

To further confirm that it was the formation of disulfide bond between C225 and C777 that stabilized the AcrB trimer and reduced the function loss caused by P223G mutation, we conducted the drug susceptibility assay in the presence of 4 mM DTT (Table 2). Our data showed that the addition of 4 mM DTT had no effect on the drug susceptibility of BW25113*ΔacrB* containing WT AcrB, AcrB_P223G_, or the empty plasmid pQE70, but specifically reduced the MIC of BW25113*ΔacrB* containing _CL_AcrB_P223G/V225C/A777C_. Membrane vesicles from the corresponding strain were extracted and analyzed using Western blot analysis without further addition of DTT. A significant portion of _CL_AcrB_P223G/V225C/A777C_ trimer was reduced to monomers, consistent with the observed decrease of activity (data not shown).

**Table 2:** MIC (µg/ml) of BW25113*ΔacrB* containing plasmid encoded AcrB measured in the presence (no bold) or absence (bold) of 4 mM DTT.

Plasmids	Ery	Ery	TPP	TPP
pQE70-AcrB	**80**	80	**640**	640
pQE70	**2.5**	2.5	**5**	5
pQE70-AcrB_P223G_	**5**	5	**20**	20
pQE70-_CL_AcrB_P223G/V225C/A777C_	**40**	20	**80**	20–40

## Discussion

In an effort to understand the AcrB trimerization process and the inter-subunit interactions that lead to trimerization, we have previously developed a disulfide trapping method to characterize AcrB tertiary structure in membrane and created a well folded monomeric AcrB mutant. In this study, we investigated the role of a protruding loop during AcrB trimerization by studying the effect of mutating a conserved residue, P223. Based on the crystal structure of AcrB, each subunit contains a protruding loop, which extends deep into a tunnel in the neighboring subunit. The loop-to-tunnel interaction is apparently very important to the formation and stability of AcrB trimer. The unique backbone structure of P223 induces the loop to form a kink close to the tip (Figure 1), which suggests that it may function as a wedge to lock the loop in place and stabilize the trimer structure. As expected, the mutation of P223 into other amino acids, especially Gly, drastically reduced the activity of AcrB. However, AcrB_P223G_ was different from a monomeric AcrB mutant, AcrB_Δloop_, which we created previously. AcrB_Δloop_ was completely functionless while AcrB_P223G_ still had a very low level of activity, which implied that at least a small portion of the protein should still exist as trimers *in vivo*. Subsequently, we successfully trapped trimers formed by mutant containing P223G mutation using an inter-subunit disulfide bond, C225–C777. The stability of AcrB_P223G_ trimer was apparently much weaker than that of the WT AcrB as it dissociated upon detergent extraction and purification.

It is intriguing to speculate how the interaction between the protruding loop and the corresponding tunnel in the neighboring subunit is established. We probed the inter-subunit interface between neighboring subunits in AcrB using the online server of ProtorP [36]. When AcrB trimerizes, the loop-and-tunnel interaction between neighboring subunits contributes approximately 1,600 Å^2^ of decreased accessible surface area (ASA), which is 46.9% of the overall inter-subunit interface. Studies have shown that protein-protein interactions with interfaces larger than ∼1000 Å^2^ are likely to undergo conformational changes upon binding [37], [38]. There are three possible scenarios: the loop adopts its final structure first while the tunnel retains a certain degree of flexibility and folds around the loop (loop first); the tunnel adopts its final structure first while the loop retains a certain degree of flexibility and folds once it settles inside the tunnel (tunnel first); or both loop and tunnel are flexible and induce each other to fold into the final conformation. To investigate the flexibility of the loop during trimerization, we created a reporter Cys-pair in the loop, C216–C234. We found that this pair of Cys formed disulfide bond in AcrB, and the introduction of the P223G mutation had no effect on the formation of the bond, indicating that the P223G mutation did not affect the conformation of the loop. In addition, the formation of disulfide bond between C216–C234, which greatly restricted the flexibility of the loop, had no effect on the drug efflux activity when introduced into the fully functional Cys-less AcrB background (Table 1). Assuming disulfide bond forms when the subunit acquires its tertiary structure, prior to trimerization, these results would suggest that the flexibility of the loop structure is not critical for trimerization. This assumption is reasonable, as studies have shown that intra-molecular disulfide bond in proteins formed on the time scale of sec to min, comparable to the time it takes to translate a polypeptide chain with the size of AcrB (1049 amino acid, assuming a translation rate of 10–15 amino acids per second) [39], [40]. In many cases the formation of disulfide bond is actually coupled with protein folding [41]. These results suggested that the loop remained rigid during trimerization. Another line of evidence that support the “loop-first” mechanism is the observation that the activity of AcrB_P223Y_ is comparable or slightly higher than that of AcrB_P223G_. If the tunnel forms first, a loop with such a large side chain at the tip would have trouble penetrating through the tunnel. If the loop remains rigid during the trimerization process, then the introduction of a Pro to Gly mutation may have caused the loop to become more flexible, which would result in an increased entropy penalty when the loop adopts a specific structure upon trimerization. However, entropy penalty itself cannot completely account for the significant loss of function in AcrB_P223G_, since the P224G mutation had no obvious effect on protein function. P223 could be serving as a wedge at the tip of the loop, which prevented the loop from slipping out of the tunnel once the trimer formed.

A random mutagenesis study on a close homologue of AcrB, MexB in *Pseudomonas aeruginosa*, has lead to the identification of a functionally defective mutant with a point mutation in the loop, G220S [42]. G220 is another conserved residue in the loop (Figure 1D and 2). The authors found that the mutant expressed at a level similar to that of WT MexB, and speculated that the G220S mutation may have hindered the insertion of the loop into the tunnel and thus decreased efficiency of MexB trimer formation through changing the secondary structure of the loop and causing steric problems. The position of the corresponding G220 in AcrB structure locates right at the kink in the loop (Figure 1D), which further confirmed the importance of the kink in stabilizing the trimer structure. The loop locates right in between the TolC-docking domain and pore-forming domain of AcrB. There is also a possibility that in P223G mutation, transport-dependent conformational change could have been affected, which may further contribute to the observed drastic decrease of activity.

From an evolutionary perspective, protein oligomerization offers clear functional advantages including enhanced structural scaffolding to support and regulate function, increased sensitivity to evolutionary pressure, and improved stability [43]–[45]. However, the exact mechanisms by which proteins assemble into oligomers remain poorly understood. Here we used a homo-trimeric membrane protein, AcrB, as a model system and investigated the connection between the oligomer stability and protein activity. We found the mutation of a residue critical to inter-subunit interaction “loosened” the AcrB trimer and thus drastically decreased the transport activity of the efflux pump. When tightened using an inter-subunit disulfide bond, the activity of the mutant improved dramatically. In addition, our result showed that during the trimerization of AcrB, the long protruding loop remained rigid, which suggested that its binding partners in the neighboring subunit underwent conformational adjustment to form a tunnel to accommodate the loop.

## Materials and Methods

### Materials

Protein molecular weight markers for SDS-PAGE and BN-PAGE were from Fermentas (Glen Burnie, MD) and Invitrogen (Carlsbad, CA), respectively. The custom polyclonal rabbit anti-AcrB was ordered from GenScript (Piscataway, NJ), produced following a protocol as described [18]. All enzymes were from New England Biolabs (Ipswich, MA). The parent WT (BW35113) and *acrB* knockout strain (BW35113Δ*acrB*) strains were obtained from the Yale *E. coli* genetic stock center. AG100A is a kind gift from Dr. Hiroshi Nikaido.

### Site-Directed Mutagenesis, Expression and Purification of AcrB and its Mutants

Mutations were introduced into the *acrB* gene in plasmid pQE70-AcrB using the QuikChange method (Agilent Technologies). Protein expression and purification was conducted as described [32].

### Drug Susceptibility Assays

Drug susceptibility was determined by two methods. The MICs of erythromycin, novobiocin, rhodamine 6G, tetraphenylphosphonium, and tetracycline as shown in Table 1 were determined on agar plate without induction by the two-fold agar dilution method [46]. Cells harboring pQE70-AcrB derived plasmids were incubated on the plate at 37°C for 20 h and growth was evaluated. To detect the effect of DTT, the drug susceptibilities experiments were conducted in liquid cultures as described [18]. Briefly, the exponential-phase cultures started from a single colony of freshly transformed cells was diluted to a final OD_600_ of 0.1 unit with LB broth. 10 µl of this dilute culture was used to inoculate each well of a sterile 24-well culture plate containing 1 ml fresh LB medium. The plate was incubated at 37°C with shaking at 200 rpm for 8 hours before the OD_600_ was measured. Each assay was repeated at least three times.

### Expression Level Analysis Using Western Blot

BW25113*ΔacrB* cells transformed with plasmids encoding AcrB or its mutants were cultured at 37°C overnight. The cells were harvested, resuspended in sodium phosphate buffer (pH 7.4) buffer, and lysed using a French press. Cell debris was removed through a low-speed centrifugation and membrane vesicles were collected by ultracentrifugation at 150,000 g for 1 h at 4°C. Membrane vesicles were solubilized in sodium phosphate buffer (pH 7.4) containing 2% (wt/vol) SDS at room temperature and separated on a 8% SDS-PAGE gel. The proteins were transferred to a nitrocellulose membrane (Millipore, Bedford, MA) and detected as described [20].

### CD Spectroscopy, Disulfide Trapping, and Blue Native (BN)-PAGE Analysis

CD spectra and temperature denaturation scans were collected on a JASCO J-810 spectrometer as described [20]. Disulfide trapping was performed as described [32]. BN-PAGE was performed as described [20].

### Limited Trypsin Digestion

Trypsin was mixed with WT AcrB or AcrB_P223G_ at the mass ratio of 1∶200 in buffer (10 mM sodium-phosphate, 50 mM NaCl, 10% glycerol, 0.05% n-Dodecyl β-D-maltoside (DDM), pH 7.5) at room temperature. At indicated time, aliquots of sample were withdrawn, quenched through the adding of SDS loading buffer and phenylmethylsulfonyl fluoride (PMSF), and put on ice. The samples were resolved on SDS-PAGE.
